# Perforated Duodenal Diverticulum with Subtle Pneumoretroperitoneum on Abdominal X-Ray

**DOI:** 10.1155/2017/7089573

**Published:** 2017-10-19

**Authors:** Yuzeng Shen, Mark Kwok Fai Leong

**Affiliations:** Department of Emergency Medicine, Singapore General Hospital, Singapore

## Abstract

Abdominal pain is one of the most common presenting complaints at the Emergency Department (ED). Given the myriad of possible differential diagnoses for abdominal pain, it becomes more important to diagnose conditions requiring emergent surgical intervention early. We present a case of an elderly male patient with abdominal pain secondary to perforated hollow viscus, subtle evidence of pneumoretroperitoneum on the initial supine abdominal X-ray, and review the signs of pneumoperitoneum and pneumoretroperitoneum on plain abdominal X-rays.

## 1. Introduction

Perforated hollow viscus may result in pneumoperitoneum or pneumoretroperitoneum, depending on the location of perforation along the gastrointestinal tract [1, 2], and may lead to significant mortality and morbidity. X-rays are often the first-line radiological investigation at the ED to assess for extraluminal air in the abdomen suggestive of gastrointestinal perforation; however, despite being the initial investigation of choice, X-rays have a sensitivity of 50–70% in detecting extraluminal air within the abdomen [2, 3]. We present a case of an elderly patient with a perforated duodenal diverticulum and subtle evidence of pneumoretroperitoneum on the initial supine abdominal X-ray.

## 2. Case Presentation

An 85-year-old male presented to the ED with complaints of constipation with no passage of flatus, generalized abdominal discomfort, and abdominal distension for 4 days. His symptoms were progressively worsening over the 4 days. There was a single episode of nonbilious vomiting on the day prior to his ED visit. He had been taking oral tramadol tablets (dose: 50 mg TDS PRN) for pain relief after sustaining a vertebral fracture a month ago. The abdominal discomfort, constipation, and vomiting episode may have been associated with consumption of tramadol tablets. There were no prior episodes of similar symptoms and no identified relieving factors. He did not have any other associated symptoms such as fever, change in bowel habits, or history suggesting gastrointestinal bleeding.

His vital signs were as follows: temperature 35.7°C; pulse rate 79 beats per minute; respiratory rate of 17 per minute; blood pressure 125/59 mmHg; pulse oximetry reading of 99% on room air; and pain score of 2/10. On physical examination, the patient was alert and conversant. Hydration was good and he did not appear pale or jaundiced. On abdominal examination, the abdomen was soft without any focal tenderness, there was no rebound, guarding or palpable abdominal mass, abdominal distension was noted, and bowel sounds were present and normal sounding. Renal punch was negative. On digital rectal examination, rectum was empty. Anal tone and perianal sensation were intact.

Full blood count showed slight elevation of white blood cell count 12.6 × 10^9^/L, with neutrophil predominance. Renal panel showed acute renal impairment with elevated urea (18 mmol/L), creatinine (266 umol/L), and decreased bicarbonate (15.9 mmol/L). Laboratory tests were otherwise unremarkable.

**Figure 1:**
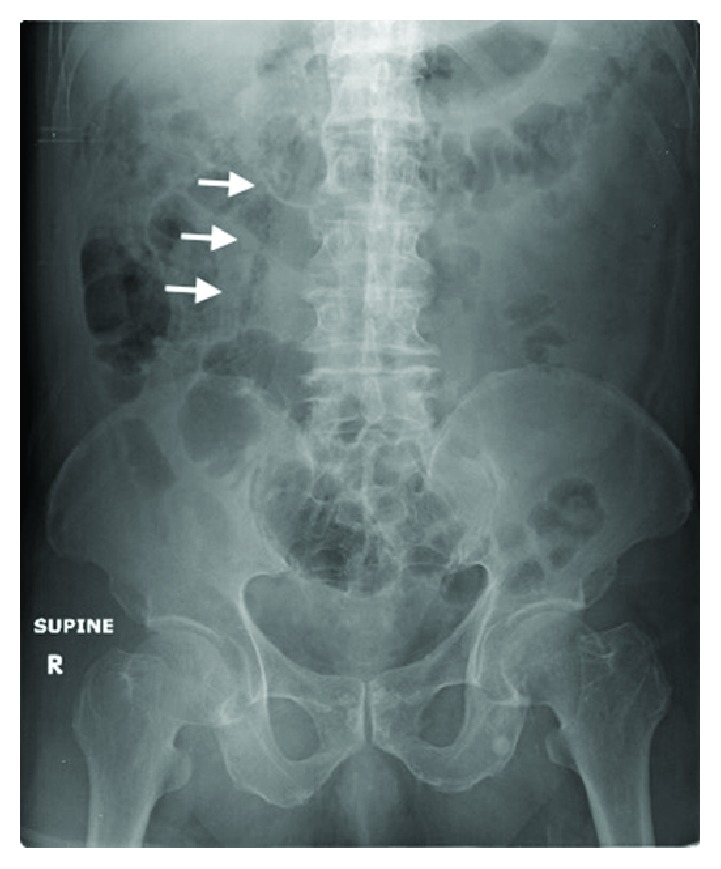
Abdominal X-ray showing lucency outlining the right crus of the diaphragm and extending along the right psoas muscle.

Erect chest X-ray showed no free air under diaphragm. Supine abdominal X-ray showed no dilated bowel loops, but there was an abnormal lucency at the right abdomen outlining the right crus of the diaphragm and extending along the right psoas muscle (Figure 1). The findings on abdominal X-ray were noted after the patient was transferred to the inpatient ward for further management.

CT scan of the abdomen and pelvis was ordered at the inpatient setting for the patient in lieu of the findings on abdominal X-ray. The physical examination findings progressed with time and the patient developed generalized abdominal tenderness and guarding over the epigastrium and right flank region around 11 hours after his initial presentation to the ED. A CT scan of the thorax was also subsequently done to exclude esophageal perforation.

**Figure 2:**
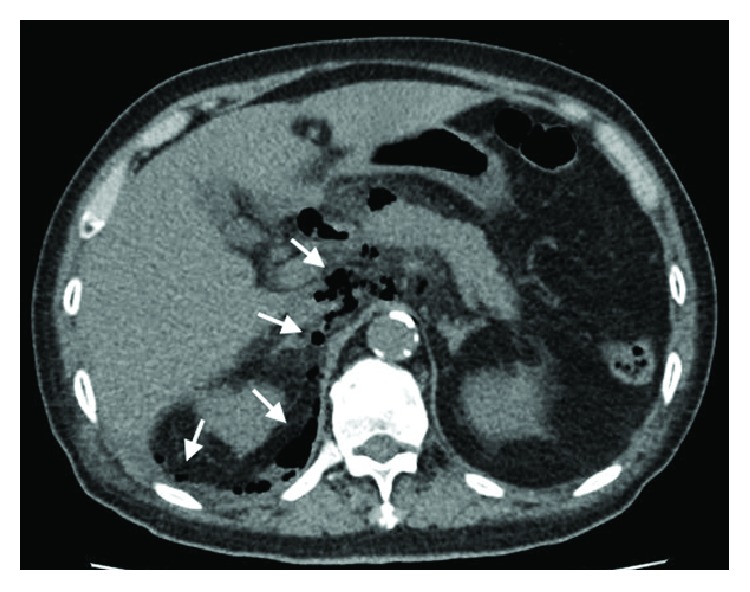
CT scan of abdomen at the level of the pancreas showing pneumoperitoneum with retroperitoneal air involving the right perinephric region.

**Figure 3:**
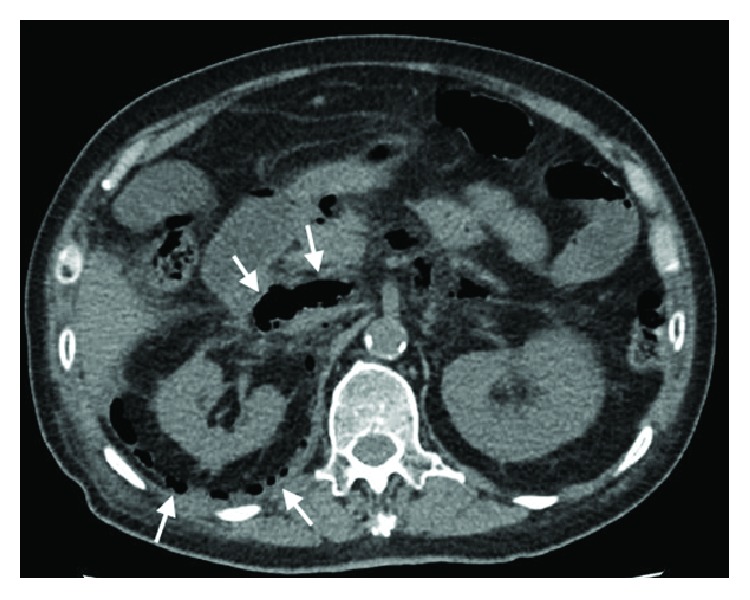
CT scan of abdomen showing pneumoperitoneum at region of 2nd and 3rd part of the duodenum with retroperitoneal air involving the right perinephric region.

**Figure 4:**
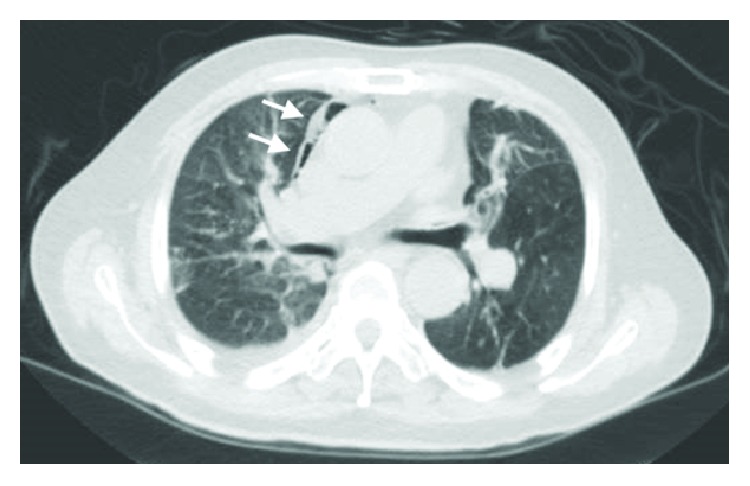
CT scan of thorax illustrating presence of pneumomediastinum at the level of the aortic arch.

**Figure 5:**
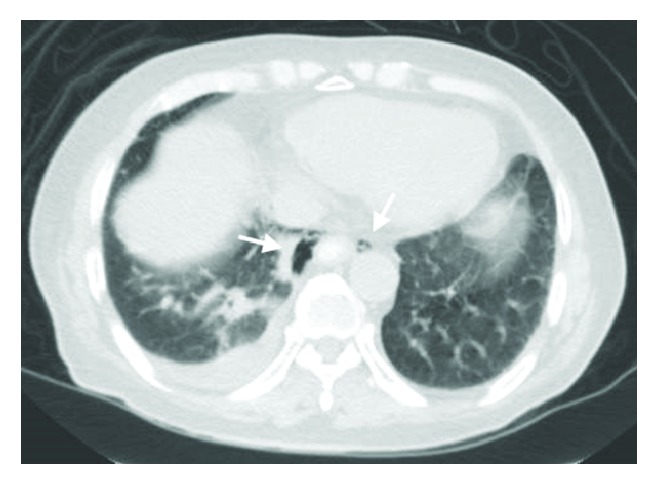
CT scan of thorax illustrating presence of pneumomediastinum at the level of the diaphragm.

CT imaging done showed extensive retroperitoneal gas with mediastinal extension (Figure 2) and there was radiological suspicion of perforation at the region of the 2nd and 3rd part of the duodenum (Figure 3). There was pneumomediastinum identified on CT scan of the thorax (Figures 4 and 5). The patient was subsequently diagnosed to have perforated duodenal diverticulum during laparotomy, for which a partial duodenal resection and duodenojejunostomy was performed. The etiology of hollow viscus perforation was unable to be ascertained despite CT imaging and surgical intervention.

## 3. Discussion

Besides perforated hollow viscus, other more benign causes of pneumoperitoneum include recent abdominal surgery, endoscopic procedures, and peritoneal dialysis [4]. The incidence of pneumoperitoneum secondary to perforated hollow viscus ranges from 80% to 91%, of which peptic ulcer disease contributes more than 90% [1, 2]. Other possible causes of hollow viscus perforation include consumption of medications such as steroids and NSAIDs which predispose to perforation, foreign body ingestion, mechanical trauma from instrumentation of the gastrointestinal tract, and underlying malignancy [5].

Signs of peritonism on clinical examination develop as gastrointestinal contents from the perforation irritate the peritoneum, leading to findings such as guarding and rebound tenderness of the abdomen. When the perforation occurs within and remains restricted to the retroperitoneal space, the presentation may be subtle and atypical, with possible complaints of associated back pain. As with this case presentation, there may not be typical findings of abdominal tenderness or peritonism during the initial stages when pneumoretroperitoneum remains localized.

Signs of pneumoperitoneum on abdominal X-ray are more often discussed and highlighted, compared to those signs associated with pneumoretroperitoneum. They include Rigler's sign, hyperlucent liver sign, doge cap sign, falciform ligament sign, urachus sign, football sign, cupola sign, and air under diaphragm [6]. Studies have shown that the sensitivity of using the supine abdominal X-ray to detect pneumoperitoneum ranges from 56% to 80.4% and associated with a false positive rate of 13% [1, 7–9].

The development of positive physical findings on clinical examination was preceded by subtle presentation of pneumoretroperitoneum on abdominal X-ray, hence the importance in recognising the features suggestive of pneumoretroperitoneum on plain radiographs. In patients with initial diagnosis which are indeterminate, serial abdominal examination remains helpful to identify evolving abdominal pathology [10], especially for patients who may present in a subtle manner or atypically.

**Figure 6:**
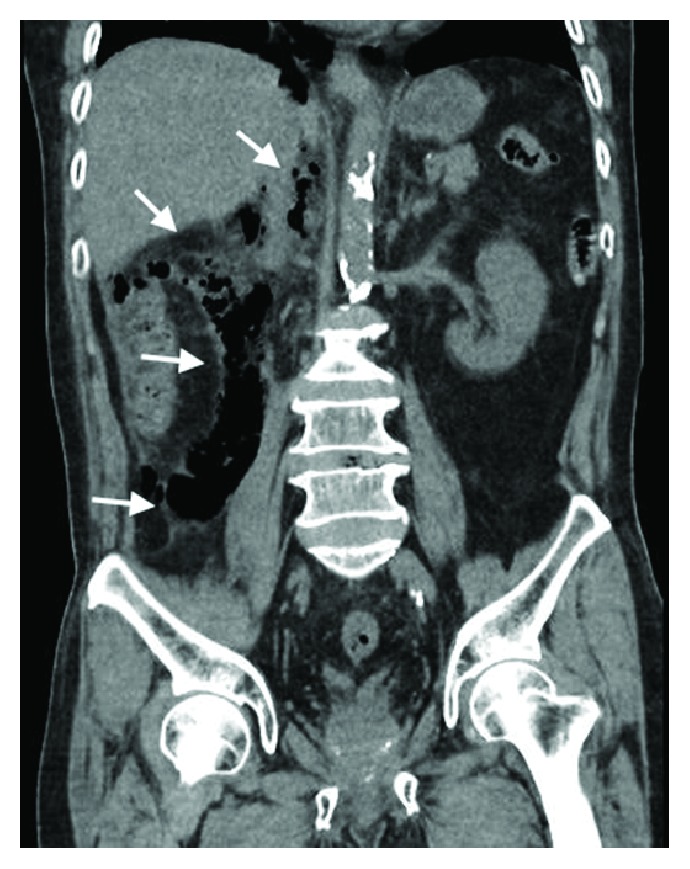
CT scan of abdomen illustrating extraluminal air outlining the right kidney, psoas muscle, and diaphragmatic crus.

The patient presented with free air in the retroperitoneal space, which communicated superiorly with the posterior mediastinum via the diaphragmatic aortic hiatus and inferiorly to the right iliac fossa at the level, above the right inguinal ligament. Radiological features on X-ray suggestive of pneumoretroperitoneum include air outlining the kidney, psoas shadow, and diaphragmatic crus, usually more common on the right. There may be presence of mediastinal or cervical emphysema [11–13]. The features described are more obvious on CT imaging in this case presentation (Figure 6).

This case highlights the difficulties in diagnosing pneumoretroperitoneum with a supine abdominal X-ray. ED physicians should be familiar with subtle signs of extraluminal air on the abdominal X-ray to avoid delayed diagnosis of a potentially high morbidity and mortality condition.
